# Normal macrophage signaling and gene expression in *Rosa26* Cas9-expressing mice

**DOI:** 10.1093/immhor/vlaf047

**Published:** 2025-10-09

**Authors:** Monika D Hermann, Nuria Fernandez Perez, Assa Yeroslaviz, Peter J Murray

**Affiliations:** Max Planck Institute of Biochemistry, Martinsried, Germany; Max Planck Institute of Biochemistry, Martinsried, Germany; Max Planck Institute of Biochemistry, Martinsried, Germany; Max Planck Institute of Biochemistry, Martinsried, Germany

**Keywords:** Crispr/Cas9, macrophage, macrophage polarization, Rosa26, signaling

## Abstract

Cas9-expression from the *Rosa26* “safe harbor” locus are widely used for gene manipulation and Crispr-based screening. Recently, experimental evidence suggested that macrophages isolated from *Rosa26*-Cas9 mice may have signaling differences compared to control mice in terms of TRIF signaling downstream of TLR3 and TLR4. As we frequently use the *Rosa26*-Cas9 mice made by Feng Zhang (Cas9-FZ, Jackson Laboratory stock No. 026179), arguably the strain with the widest distribution and utilization, we were motivated to test macrophage signaling in these mice under our conventional conditions. We used different macrophage polarization and signaling conditions combined with RNA sequencing and measurement of TLR signaling by immunoblotting. Our results suggest that the Cas9-FZ mice bear no obvious defects in any commonly used macrophage signaling pathway. We document the differences in our macrophage culture techniques compared to Raychowdhury et al., which may aid in how individual laboratories use Cas9-expressing macrophages, especially for focused or genome-wide screening.

## Introduction

Raychowdhury et al. performed extensive experimentation with different Cas9-expressing bone marrow–derived macrophages (BMDMs).^1^ Their conclusions centered on defects in IRF3 activation, TLR3/4 signaling, and NF-κB activation following LPS or poly I: C stimulation.^1^ Because the use of Cas9-expressing mice is now a pillar of modern experimentation, we were strongly motivated to check the behavior of BMDMs isolated from the Feng Zhang Cas9 mice (B6J.129(Cg)-Gt(ROSA)26Sor^tm1.1(CAG-cas9*,-EGFP)Fezh^/J) (Jackson Laboratory strain No. 026179, hereafter called Cas9-FZ), which is a widely used and distributed strain.^2^ In our colony and in the way we generate BMDMs, some differences exist compared to the carefully documented report of Raychowdhury et al.^1^ Nevertheless, we were surprised at the results Raychowdhury et al. reported and, therefore, undertook our own investigation into the behavior of BMDMs from the Cas9-FZ mice. Our approach was distinct from Raychowdhury et al. in that we took a broader approach to macrophage signaling and “polarization” to understand and document any defects as opposed to a highly focused investigation of TRIF-linked signaling. Here, we report our findings and further elaborate on the differences in experimental approaches.

## Materials and methods

### Mice, mice housing, and breeding

Our strain of Cas-FZ mice was originally a gift of Dr Christian Mayer in our sister institute, the Max Planck Institute of Biological Intelligence. In our shared animal facilities, movement of strains between buildings requires rederivation by policy. In addition, our institutes uniformly use the C57BL/6N strain (eg as stock controls and for embryo transfer and other genetic manipulations) as opposed to the C57BL/6J background. These substrains have several key differences documented on the Jackson Laboratory website (https://www.jax.org/news-and-insights/jax-blog/2013/october/three-ways-a-b6j-mouse-differs-from-b6n-and-why-it-should-m). Our mice are housed in an open cage format in 2 large rooms with a 14:10 light cycle and ad libitum food and water (from bottles). All mice in our colony are *Helicobacter*-free and monitored for infections according to the hygiene policy of the Institute.

### Genotyping and allele selection

The Cas9-FZ mice were genotyped according to the information supplied on the Jackson Laboratory website. In our colony, we generally use mice homozygous for the *Rosa26* knock-in allele (ie FZ/FZ). In some experiments, we used heterozygous mice (FZ/+).

### Macrophage culture and stimulation

BMDM preparations were performed according to established protocols in use in our laboratory for 2 decades.^3^^,^^4^ In brief, and in distinction to Raychowdhury et al.,^1^ we begin with 150-mm tissue culture dishes where the bone marrow from femurs and tibias from one mouse is plated to one to three 150 mm in 30- to 40-mL media dishes depending on the final cell number needed. We use conventional DMEM (Thermo Fisher Scientific, 41966-029) with 10% FBS (Biochrome, S0115) and 1% penicillin-streptomycin (VWR, 392-0406) as the culture media as opposed to RPMI 1640 in Raychowdhury et al. The reason we use DMEM is that the lack of amino acids (L-alanine, L-asparagine, L-aspartic acid, L-glutamic acid, and L-hydroxyproline) slows the growth rate of macrophage progenitors relative to the culture in RPMI-type media, which contains all 20 proteinaceous amino acids. The empirically determined slower growth rate enhances the number of “floating” CSF1-dependent progenitors and results in a superior final yield of adherent macrophages at day 7 of culture, especially when pulsed with fresh CSF1 on days 3 and 5 of the culture. An additional difference is our use of highly purified recombinant human CSF1, which we express in 293T cells. In-house–generated human CSF1 enables us to add more cytokine to increase cell yield as needed. Human CSF1 binds to and signals via the murine CSF1 receptor.^5^ We noted that Raychowdhury et al. additionally added minimal nonessential amino acids to their RPMI 1640 media along with β-mercaptoethanol. Raychowdhury et al. did not state their cell collection method; in our case, we universally scrape the cells using Sarstedt cell scrapers, scraping the bevel away from the body at ∼45°. Our stimulation conditions used IL4 + IL13 (both at 10 ng/mL) and LPS + IFN-γ (L4391, Sigma, at 5 ng/mL; 315-05, PeproTech at 2 ng/mL) as indicated in the figure legend. CpG ODN 1826 was purchased from Integrated DNA Technologies (Leuven, Belgium) and used at 2 µM. We generated our own murine IL-4 in baculovirus expression systems and IL-13 was purchased from PeproTech (210-13-50).

### Antibodies

Abs used for immunofluorescence were pSTAT1^(Tyr701)^ (Cell Signaling Technology, 7649), STAT1 (Santa Cruz Biotechnology, sc-346), pIRF3 ^(S396)^ (Cell Signaling Technology, 4947S), IRF3 (Cell Signaling Technology, 4302S), pTBK1 ^(S172) XP^ (Cell Signaling Technology, 5483T), TBK1/NAK (Cell Signaling Technology, 3504S), and Vinculin (Cell Signaling Technology, 13901S).

### Bulk mRNA sequencing

BMDMs were plated in triplicates in a 6-well format. Cells were activated for 24 hours with either IL4 + IL13 or LPS + IFN-γ as described above, and DMEM was used as a control. For RNA isolation, cells were lysed in 1 mL TRIzol reagent transferred into a 1.5-mL tube, and frozen at −80 °C overnight. The following day, samples were thawed on ice, and 200 µL chloroform was added. Samples were mixed and centrifuged at 18,000 × *g* at 4 °C for 10 minutes before the colorless phase was transferred into a fresh 1.5-mL tube containing 500 µL 100% isopropanol. After RNA precipitation at −20 °C overnight, samples were centrifuged, and the RNA pellets were washed with 70% ethanol. Following the last centrifugation step and ethanol removal, pellets were air dried for ∼5 minutes before 25 µL of nuclease-free water was added.

Bulk mRNA sequencing libraries were prepared with 250 ng of total RNA of each sample using the NEBNext Ultra II Directional RNA Library Prep Kit for Illumina (NEB, E7760) with NEBNext Poly(A) mRNA Magnetic Isolation Module (NEB, E7490), according to the manufacturer’s standard protocol. Total RNA and the final library quality controls were performed using Qubit Flex Fluorometer (Thermo Fisher Scientific, Q33327) and 4200 TapeStation System (Agilent, G2991BA) before and after library preparation. The libraries were sequenced on Illumina NovaSeq 6000 SP flow cell (2 × 60 bp) and demultiplexed by bcl2fastq Conversion Software (Illumina).

After checking the samples’ quality (FastQC v.0.12.1), the files were mapped against the mouse genome (Genome build *GRCm38*) downloaded from Ensembl using the star aligner (v.2.7.10b) and default parameters. The mapped files were then quantified on gene level based on Ensembl annotations, using the featureCounts (v.2.0.4) from the SubRead package, discarding multioverlapping reads. Using the DESeq2 package (R 4.3.2, DESeq2 v.1.42), the count was normalized by the size factor to estimate the effective library size. A filtering step for removing genes with <10 reads in at least 3 samples was used. Genes with an adjusted *P* value of ≤0.01 were then considered to be differentially expressed for downstream analysis. After calculating the gene dispersion across all samples, the comparison of the 2 different conditions resulted in a list of differentially expressed genes.

## Results

### RNA sequencing

Raychowdhury et al.^1^ found that LPS stimulation of Cas9-FZ BMDMs (we assume they used homozygous mice, ie FZ/FZ) was substantially different to control mice in terms of both quality and quantity of mRNAs. Raychowdhury et al. noted that hundreds of mRNAs were deregulated (predominantly downregulated) relative to controls.^1^ Ergo, we repeated this form of experiment using our approaches but focusing on commonly used “M1” or M(LPS + IFN-γ) and “M2” or M(IL4 + IL13) because these are the most frequently used stimuli to generate large distinctions in macrophage phenotype and function.^6^^,^^7^ First, we verified that stimulation with LPS + IFN-γ or IL4 + IL13 produced the expected effects in wild-type (WT) BMDMs (Fig. S1). Indeed, canonical “M1” and “M2” transcripts were induced as expected from the extensive literature on this topic.

**Figure 1.**
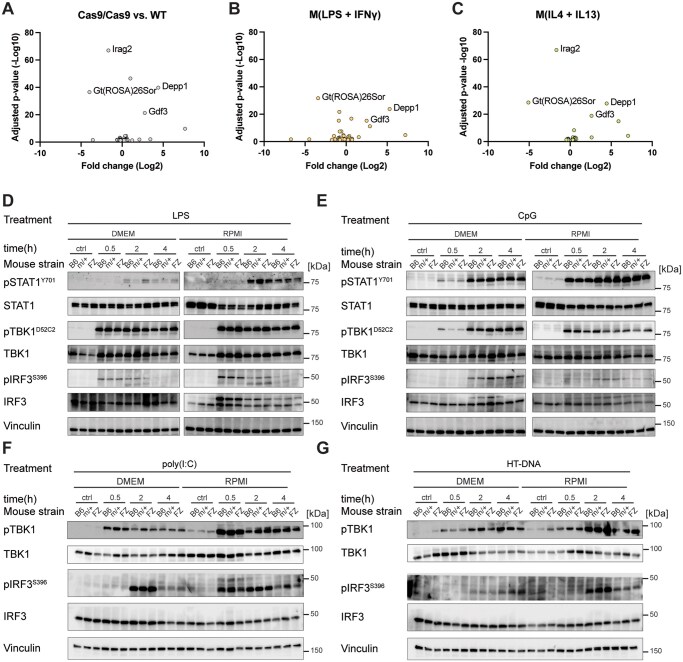
Comparative gene expression in WT and FZ/FZ BMDMs. BMDMs were generated from WT or FZ/FZ mice and were left unstimulated. (A) Stimulated with LPS + IFN-γ. (B) To the “M1” state or IL4 + IL13 to the “M2” state. (C) RNA-seq was then performed on 3 biological replicates. Transcripts common to both WT or FZ/FZ mice cancel and the residual differentially expressed mRNAs are shown as a volcano plot. Gt(ROSA)26Sor indicates the main detected transcript from the *Rosa26* locus. (D–G) Stimulation of macrophages from WT, heterozygous (m/+), or homozygous FZ BMDMs generated in DMEM or RPMI over time, where key TLR pathway intermediates were measured by immunoblotting.

Using the same system, we next compared unstimulated, M(LPS + IFN-γ) or M(IL4 + IL13) macrophages from WT versus FZ/FZ BMDMs. Our results indicated very few mRNA differences between the macrophages in any condition (Fig. 1A–C). Importantly, the most downregulated transcript in each condition originated from the Rosa26 locus as expected, as the Cas9-FZ knock-in eliminates the normal transcripts from both alleles. We observed a small number of other dysregulated transcripts (Irag2, Depp1, Gdf3) in the FZ/FZ BMDMs in each condition. While our first thought was that these mRNAs might be linked to the *Roas26* locus, in fact they originated from different chromosomes. In our experiments, the effects of LPS + IFN-γ or IL4 + IL13 “zero” out in the comparative analysis. In this case, we should have expected substantial changes in transcript profiles following Raychowdhury et al.^1^ Instead, the handful of transcripts altered, none of which have an obvious link to macrophage biology or inflammation, argues that the FZ/FZ BMDMs have an overtly normal response to LPS + IFN-γ or IL4 + IL13.

### TLR signaling

We next measured a selection of downstream signaling events from TLR3, TLR4, and TLR9 (Fig. 1D–G). In these experiments, we compared BMDMs generated in DMEM versus RPMI 1640 (with stimulations being performed in the same media as used for expansion and differentiation). In addition, we compared WT and FZ/FZ homozygous mice with heterozygous FZ/+ mice. We measured activation of pSTAT1, pTBK1, and pIRF3 at 0, 0.5, 2, and 4 hours of stimulation with LPS or CpG. Consistent with the RNA-seq findings, we did not observe any substantial differences between genotypes (Fig. 1D–G). However, we noted some distinctions in pathway activation kinetics and strength between DMEM and RPMI. Whether these differences are biologically meaningful remains unclear. Regardless, we observed no obvious differences in the responses of macrophages between genotypes, which was our central question.

## Discussion

We conclude that the Feng Zhang Cas9 Rosa26 knock-in mice^2^ are sufficiently similar on macrophage activation to WT controls that Crispr experiments can be conducted with confidence. There are several limitations of our work compared to the comprehensive study of Raychowdhury et al.^1^ First, we did not investigate TLR signaling beyond conventional macrophage stimulation experiments, nor did we perform as detailed an analysis of pathway activation compared to Raychowdhury et al.^1^ Instead, our RNA-seq experiments indicated the changes at *Rosa26* resulted in a handful of changes to noninflammatory transcripts; these results stand in contrast to the alterations in hundreds of transcripts in Raychowdhury et al.^1^ A further limitation is that we focused on only one, albeit the most widely used, Cas9 knock-in strain, while Raychowdhury et al. examined 5 additional strains where Cas9 is introduced into the mouse genome in different configurations. To exactly match our findings with Raychowdhury et al., extensive research would be required, which we consider unnecessary, at least from our perspective. We note that Raychowdhury et al. used murine CSF1 while we use highly pure human CSF1. However, the CSF1 is unlikely to be the source of the differences between studies observed herein, as both groups had macrophages to work with. Thus, the reason(s) behind the differences is unclear at present.

A further important consideration is that lentiviral introduction of guide RNAs permits Crispr-Cas9–based screening of different tissue macrophages such as Kupffer cells or intestinal macrophages. Our stance is that an simple RNA-seq experiment should be performed at the beginning of the experiment to ensure that macrophages from the Cas9 Rosa26 knock-in mice reflect control macrophages.

Finally, we note that the *Rosa26* locus, originally identified in 1991 and then used as for constitutive expression “safe harbor” for insertion of genes and other genetic manipulations, assumes there are no obvious phenotypes associated with its modification(s).^8^^,^^9^ Such an assumption must be tempered by the fact that *Rosa26* has been retained through evolution, suggesting it has one or more functions. Perhaps the small number of transcripts changed in our RNA-seq data are linked to the absence of the noncoding transcripts that originate from the *Rosa26* locus function in the FZ/FZ mice?

## Supplementary Material

vlaf047_Supplementary_Data

## Data Availability

The RNA-seq data presented in the article have been submitted to the Gene Expression Omnibus under accession number GSE298884 and are also available in a processed Excel file upon request. All immunoblotting source data are available upon request.
