# Supplementary material for: Normal macrophage signaling and gene expression in Rosa26 Cas9-expressing mice
Source: Immunohorizons. 2025 Oct 9;9(11):vlaf047. doi: 10.1093/immhor/vlaf047 (PMC12597891; doi:10.1093/immhor/vlaf047)
Supplement: vlaf047_Supplementary_Data [file vlaf047_supplementary_data.docx]

**** ****

**Supplemental Figure 1.** BMDMs were generated from WT mice and stimulated with LPS + IFNγ (**left**) to the “M1” state or IL4 + IL13 to the “M2” state (**right**). Following mapping of transcripts to the mouse genome, each stimulation condition was compared to unstimulated BMDMs and the results plotted as volcano plots. Regulated transcripts typical of the macrophage polarization state are indicated.
